# Supplementary material for: Jumping to conclusions, general intelligence, and psychosis liability: findings from the multi-centre EU-GEI case-control study
Source: Psychol Med. 2020 Apr 24;51(4):623–33. doi: 10.1017/S003329171900357X (PMC8020493; doi:10.1017/S003329171900357X)
Supplement: Supplementary file 1 [file S003329171900357Xsup001.docx]

**Supplementary Table S1. Binary mediation model results**

| DV=JTCyes/no | Coeff/SE | 95% CI | P value | R^2^ |  |
| --- | --- | --- | --- | --- | --- |
| *Model 1* | | | | 9% |  |
| IV=Case/control | 0.7/0.1 | 0.5 to 0.9 | <0.001 |  |  |
| *Model 2* | | | | 13% |  |
| MV=IQ | -0.03/0.003 | -0.04 to -0.03 | <0.001 |  | |
| IV=Case/control | 0.3/0.1 | 0.04 to 0.5 | 0.019 |  |  |
|  | | | | |  |
|  | Boot indirect | Boot SE | Boot CI^a^ | |  |
| Indirect effect | 0.1 | 0.01 | 0.1 to 0.2 | |  |

*Note.* DV=dependent variable; IV=independent variable; MV=mediator variable; JTC=jumping to conclusions; IQ=intelligence quotient

a. 95% bootstrap (N=5000) confidence interval reported.

**Supplementary Table S2. Logistic Regressions of Polygenic Risk Scores Predicting JTCyes/no**

| ​ | Odds Ratio​ | P value​ | 95% CI​ | R^2^​ |
| --- | --- | --- | --- | --- |
| Case vs Control^a^ | ​1.6 | ​<0.001 | ​1.3 to 2.1 | 10%​ |
| SZ PRS^a^ | 1.2 | 0.264 | 0.9 to 1.7 | 9% |
| SZ PRS^b^ | ​1.02 | ​0.929 | ​0.7 to 1.4 | 10%​ |
| SZ PRS^c^ | ​0.9 | ​0.844 | ​0.7 to 1.4 | 14%​ |
| IQ PRS^a^ | 0.8 | ​<0.001 | 0.7 to 0.9 | 11% |
| IQ PRS^b^ | ​0.8 | ​0.001 | ​0.7 to 0.9 | 11%​ |
| IQ PRS^c^ | ​0.9 | ​0.075 | ​0.8 to 1 | 14%​ |

*Note.* SZ=schizophrenia; PRS=Polygenic Risk Score; IQ=intelligent quotient.

^a^Adjusted for age, sex, and 20 principal components for population stratification

^b^Adjusted for case/control, age, sex, and 20 principal components for population stratification

^c^Adjusted for case/control, age, sex, and 20 principal components for population stratification

**Supplementary Table S3. Logistic Regressions of Positive symptoms and Psychotic-Like Experiences Predicting JTCyes/no**

| ​ | Odds Ratio​ | P value​ | 95% CI​ | R^2^​ |
| --- | --- | --- | --- | --- |
| Case sample N=805 | | | | |
| Delusions^a^ | ​0.9 | ​0.061 | ​0.7 to 1 | 0.3%​ |
| Delusions^b^ | 1.04 | 0.671 | 0.9 to 1.3 | 18% |
| Hallucinations^a^ | ​1.2 | ​0.036 | ​1 to 1.4 | 0.4%​ |
| Hallucinations^b^ | 1.2 | ​0.112 | 0.9 to 1.4 | 18% |
| Control sample N=1286 | | | | |
| CAPE-POS^a^ | 2.6 | 0.001 | 1.5 to 4.5 | 0.7% |
| CAPE-POS^b^ | 2.5 | 0.008 | 1.3 to 4.8 | 18% |

^a^Unadjusted

^b^Adjusted for age, sex, ethnicity, IQ, country.

**Supplementary Table S4. Prevalence of CAPE psychotic experiences in population controls**

| **CAPE POSITIVE ITEM** | **Item no.** | **Valid Frequency** |
| --- | --- | --- |
| Do you ever feel as if people seem to drop hints about you or say things with a double meaning? | 2 | 53.1% (678) |
| Do you ever feel as if things in magazines or on TV were written especially for you? | 5 | 17.3% (222) |
| Do you ever feel as if some people are not what they seem to be? | 6 | 75.8% (970) |
| Do you ever feel as if you are being persecuted in some way? | 7 | 18.2% (231) |
| Do you ever feel as if there is a conspiracy against you? | 10 | 11.8% (151) |
| Do you ever feel as if you are destined to be someone very important? | 11 | 30.2% (386) |
| Do you ever feel that you are a very special or unusual person? | 13 | 38% (484) |
| Do you ever think that people can communicate telepathically? | 15 | 25.9% (331) |
| Do you ever feel as if electrical devices such as computers can influence the way you think? | 17 | 10.8% (138) |
| Do you believe in the power of witchcraft, voodoo or the occult? | 20 | 26.4% (339) |
| Do you ever feel that people look at you oddly because of your appearance? | 22 | 34.9% (444) |
| Do you ever feel as if the thoughts in your head are being taken away from you? | 24 | 20.4% (59) |
| Do you ever feel as if the thoughts in your head are not your own? | 26 | 6.9% (88) |
| Have your thoughts ever been so vivid that you were worried other people would hear them? | 28 | 9.8% (126) |
| Do you ever hear your own thoughts being echoed back to you? | 30 | 31.8% (124) |
| Do you ever feel as if you are under the control of some force or power other than yourself? | 31 | 5.1% (65) |
| Do you ever hear voices when you are alone? | 33 | 6% (77) |
| Do you ever hear voices talking to each other when you are alone? | 34 | 1.8% (23) |
| Do you ever feel as if a double has taken the place of a family member, friend or acquaintance? | 41 | 2% (25) |
| Do you ever see objects, people or animals that other people cannot see? | 42 | 3.9% (50) |
